# Comparison of abdominal computed tomography to ultrasonography in the diagnosis of biliary disease in dogs with acute abdominal signs

**DOI:** 10.3389/fvets.2025.1508705

**Published:** 2025-06-23

**Authors:** Shanna M. Marroquin, Alison M. Lee, Marc A. Seitz, Robert W. Wills, Kimberly Ann Woodruff

**Affiliations:** ^1^VET.CT, Orlando, FL, United States; ^2^Department of Clinical Sciences, College of Veterinary Medicine, Mississippi State University, Starkville, MS, United States; ^3^Department of Comparative Biomedical Sciences, College of Veterinary Medicine, Mississippi State University, Starkville, MS, United States

**Keywords:** gallbladder, cholelithiasis, mucocele, cholecystitis, cystic mucosal hyperplasia

## Abstract

**Introduction:**

Computed tomography (CT) is becoming increasingly popular for canine patients; however, limited information is available comparing its performance to ultrasonography (US) in identifying canine biliary pathology causing acute abdominal signs. The purpose of this study was to compare the diagnostic performance of CT and US in detecting canine biliary disease. We hypothesized that CT would detect various canine biliary diseases with similar accuracy comparable to US, while US would be superior to CT in evaluating the bile ducts and diagnosing cholecystitis due to the small size of these structures.

**Methods:**

In this prospective, observational study, 35 client-owned dogs presenting with acute abdominal signs and suspected biliary disease—based on physical examination, complete blood count, and serum chemistry—underwent both abdominal US and arterial and venous phase abdominal CT. Two authors reviewed the randomized, anonymized CT and US studies to measure biliary structures and evaluate for biliary pathology. Agreement between each imaging modality and the final clinical diagnoses for biliary pathology was assessed by intraclass correlation coefficients (ICCs).

**Results:**

Twenty-eight dogs had biliary pathology, and seven dogs served as controls with no evidence of biliary disease. There were variable degrees of good to perfect agreement between US and CT to identify gallbladder mucoceles, gallbladder wall mass, and cholecystitis, moderate agreement when comparing gallbladder wall thickness, and poor agreement to identify cholelithiasis.

**Conclusion:**

The findings from this study suggest that CT may be used in place of US in canine patients presenting for acute abdominal signs with concern for biliary in origin.

## Introduction

Biliary diseases are uncommon but can cause acute abdominal signs and be fatal to the canine patient. Clinical signs are generally vague and include vomiting, lethargy, anorexia, jaundice, and abdominal pain. Diagnostic imaging remains an important tool for patients presenting with acute abdominal signs, as it is often required not only to make a rapid, accurate diagnosis but also to decide whether the patient requires surgical or medical management. Traditionally, abdominal US has been the modality of choice in veterinary medicine for animals presenting with acute abdominal signs (1). This is in part due to the ability to eliminate visceral superimposition, delineate parenchymal detail, and evaluate for gastrointestinal peristalsis, in addition to being commonly available in veterinary clinics and hospitals (2). However, US is not without its limitations which include inter-operator variability and experience level, limited field of view, long study time, potential patient discomfort, poor sensitivity to identify small volume pneumoperitoneum, and potential lack of visibility of areas of interest due to overlying bowel or peritoneal gas (1). Patient size is also a factor when performing US as previous authors determined CT detected a greater number of lesions, including gallbladder wall mineralization, cholecystoliths, hepatic cysts, and masses and nodules, than US in canine patients weighing more than 25 kg (3).

Currently, US is more readily available and less expensive than CT. However, as the availability of CT with specialist interpretation increases and CT costs decrease, veterinarians will begin using this modality more to image patients presenting with acute abdominal signs. Computed tomography has the potential to serve as an alternative to abdominal US when a sonographer is unavailable. In addition, CT studies can be performed quickly by trained personnel and with radiologist interpretation on or off-site. CT has been suggested to be associated with less abdominal discomfort than US as there is no pressure on the abdomen from a transducer (1, 2). However, it is unknown whether CT is as accurate as US to diagnose biliary diseases in these patients.

The purpose of this prospective study was to compare the accuracy of CT and US in diagnosing biliary disease in dogs presenting with acute abdominal signs and suspected biliary disease based on physical examination, complete blood count, and serum chemistry findings. This information can be used to aid diagnosis and hasten decision-making in critical patients. The hypotheses were CT would allow for the detection of various canine biliary diseases including bile duct enlargement, cholelithiasis, and mucocele formation with similar accuracy to US and that the two modalities would be similar for investigation of the bile ducts and the diagnosis of cholecystitis in dogs.

## Methods

This study was a prospective, observational study completed with Institutional Animal Care and Use Committee approval. The study population consisted of client-owned dogs presenting to the small animal care services from 2020 to 2021 with acute abdominal signs suspected to be related to the biliary tract (due to clinical history, bloodwork, and/or physical examination findings) and whose diagnostic plan included an abdominal US. Inclusion criteria included patients presenting for any signs of acute abdominal disease potentially involving the biliary tract including vomiting, lethargy, anorexia, jaundice, abdominal pain, and fever. Exclusion criteria included dogs with a previous cholecystectomy and dogs unable to be sedated. Dogs were enrolled following informed owner consent. Patients enrolled in the study with signs of acute abdominal disease that were not ultimately diagnosed with biliary disease with imaging served as negative controls for comparison of the performance of CT and US.

All dogs received both an abdominal US and CT study. The patients were fasted for at least 6 h prior to the examinations. The abdominal US examination was performed by an ACVR-certified radiologist or by a diagnostic imaging resident under supervision of one prior to or following the CT examination using a GE LOGIQ S8 ultrasound machine (General Electric, Boston, MA) with a C3-10-D microconvex transducer (6–10 MHz) or a 11 L-D linear transducer (9–12 MHz) depending on body size (General Electric, Boston, MA). In some cases, exams were performed by one of the two interpreting authors, due to limitations in available personnel and patient needs. Most patients received both studies within 1 h of each other except three patients that were dehydrated upon presentation as it was deemed not safe for the patients to receive intravenous contrast agent at that time. The administration of iodinated contrast agents is contraindicated in dehydrated patients (4), in part due to side effects related to contrast-induced nephrotoxicity, which is described in people and has been reported in a dog (4, 5). These patients received intravenous fluid therapy overnight. In some cases, opioid pain medication for discomfort and/or maropitant for vomiting were administered. It is important to note that opioid administration does result in decreased biliary excretions and, in extreme cases, has mimicked biliary obstruction in the short term (6). In these cases, the contrast-enhanced CT study was performed the following morning. These patients did not undergo a second US examination. All studies were performed within 24 h the examination of the other imaging modality.

For the abdominal US, the dogs were briefly placed in dorsal or left and right lateral recumbency, depending on body size and need to shift positioning to fully evaluate the desired organs. The hair was locally clipped, and coupling gel and isopropyl alcohol were applied to provide adequate probe contact. An abdominal US was performed according to the ACVR and ECVDI consensus statement and included both still images and cine clips of the liver, gallbladder, common bile duct, duodenum, and pancreas following the consensus imaging guidelines (7). B-mode with spatial compounding and Doppler ultrasound (color and power) were used in all cases. The dogs received sedation if deemed necessary to help facilitate US study acquisition; however, most of the patients were not sedated for the US examination. The sedation drugs, dosages, and routes of administration for both US and CT were selected for each patient by their attending veterinarian and were not standardized.

Dogs were sedated and underwent a dual-phase abdominal CT examination using a Toshiba Aquilion 16-slice multi-detector scanner (Toshiba Corp, Toshiba American Medical Systems, INC., Tustin, CA). If the patients were sedated during the US examination, additional sedation was administered if deemed required to allow immobilization for the CT study. Dogs were positioned in ventral recumbency and imaged using the following protocols: isotropic 16 × 0.5 mm (for dogs < 25 kg) or 16 × 1.5 mm (for dogs > 25 kg) collimation, 100–120 kVp, 80–200 mAs, a helical pitch of 1.5, a matrix of 512 × 512, a rotation time of 500 ms, and a field of view large enough to encompass the entire abdomen. All protocols were based on patient size and determined by a board-certified radiologist overseeing radiology residents, interns, and certified veterinary technicians. Precontrast, arterial (10 s post start of injection), and venous (90 s post start of injection) series were acquired with all patients receiving a bolus via manual injection of ioversol (Optiray 320, Guerbet, Princeton, JN) at 2.2 mg/kg (704 mg I/kg body weight) or iohexol (Omnipaque 240, GE Healthcare, Marlborough, MA) at 2.2 mg/kg (528 mg I/kg body weight) via a thoracic limb intravenous catheter followed by a 2–5 mL saline flush, with the assistance of a power injector for larger patients with a flow rate of 1.5 mL/s. Arterial (10 s) and venous (90 s) post-contrast series were acquired in all patients. All images were acquired using a standard FC07 soft tissue algorithm (WW = 400, WL = 50) with variable slice thickness ranging from 1 mm to 3 mm, based on patient size (1 mm for <25 kg, 3 mm for >25 kg). Transverse, sagittal, and dorsal reconstructions were created based on volume acquisitions in all phases.

An ACVR-certified veterinary radiologist (AL) and a diagnostic imaging resident (SM) who were blinded to the imaging, cytology, and histopathology results reviewed randomized, anonymized CT images and US images and cine clips using a DICOM viewer (eUnity, Client Outlook Inc., Ontario, Canada). Enough time was allowed to pass such that the two evaluators did not clearly recall cases they had imaged previously, and all cases were anonymized prior to review. The evaluators recorded the following on CT: gallbladder wall thickness, bile Hounsfield units (HU) measured on precontrast CT images in a soft tissue window in the central portion of the gallbladder lumen with a region of interest approximately half the diameter of the gallbladder, presence or absence of hyperattenuating material within the gallbladder lumen (and whether it was gravity-dependent), ability to identify the common bile duct and intrahepatic bile ducts on post-contrast imaging, and, if applicable, diameter of the ducts. Pattern of contrast enhancement of the liver and gallbladder wall was noted if abnormal. Gallbladder size was not measured, but subjective enlargement was noted if the gallbladder looked distend and round rather than pear-shaped or appeared enlarged as compared to the adjacent liver lobes. The following US findings were recorded: gallbladder wall thickness, presence or absence of hyperechoic material within the gallbladder lumen and if it was gravity-dependent, ability to identify the common bile duct and intrahepatic bile ducts, and, if applicable, diameter of the ducts. Color Doppler was used to aid in conformation for hepatic duct identification. Gallbladder size was not recorded, but subjective enlargement was recorded if the gallbladder appeared enlarged as compared to adjacent liver or took on a more rounded shape.

Gallbladder wall, hepatic duct, and common bile duct diameter and thickness measurements were performed in regions without respiratory motion artifact on the post-contrast series, and the widest diameter of each measured structure was recorded if there was a variation of size. Measurements were performed in triplicate and averaged. Thin-slice reconstructions were chosen to reduce the effects of volume averaging, thus making measurements appear artificially larger on CT. The imaging CT and US diagnoses were recorded. For objective evaluations, the observers’ measurements were averaged. For subjective evaluations, any discrepancies were discussed until a consensus was made.

Patients were considered positive for biliary pathology on CT or US for any of the following criteria: thickened gallbladder wall (>2 mm), gallbladder wall edema (characterized by gallbladder wall thickening with a central hypoechoic or fluid attenuating band), dilated common bile duct (>3 mm duct diameter) or intrahepatic bile ducts (>2 mm duct diameter), cholelithiasis, and findings previously described to be consistent with a gallbladder mucocele (8–13). Previously reported imaging changes were used to diagnose cholecystitis, including symmetric or asymmetric gallbladder wall thickening, the presence of a double-layered gallbladder wall, and a hypoechoic gallbladder wall (14, 15).

Cholecystolithiasis was characterized as mineral attenuating, sharply marginated material identified on CT and hyperechoic, distally shadowing structures displaying twinkle artifact on US. This hyperechoic material on US was also evaluated with color Doppler to evaluate for the presence of twinkle artifact which supported the diagnosis of cholecystolithiasis. Gallbladder distension was also noted and characterized by a distend and round rather than pear-shaped gallbladder and subjective gallbladder enlargement. Gallbladder sludge was characterized as mobile hyperechoic debris on US which did not display distal acoustic shadowing. GB mass was recorded if a mass was identified within the gallbladder wall or lumen. CMH was identified as sessile, nodular, mass-forming, intraluminal, hyperechoic, or hyperattenuating and mineral attenuating material within the gallbladder as previously described on CT (8) and US (16).

US and CT imaging diagnoses were recorded based solely on the imaging in each case. A separate, final clinical diagnosis was also assigned in each case, which represented the final diagnosis for which the patient was ultimately treated or diagnosed following all testing. Final clinical diagnoses were considered positive for biliary pathology as determined by cytology, histopathology, and/or clinical diagnosis based on overall examination findings (including imaging findings and bloodwork). The final clinical diagnoses were used to compare the positive or negative presumed imaging diagnoses of CT and US.

### Statistical analysis

Agreement between each imaging modality and the final clinical diagnoses for the finding of gallbladder wall mass, gallbladder mucocele, gallbladder wall edema, cholelithiasis, cholecystitis, and cystic mucosal hyperplasia (CMH) was assessed by intraclass correlation coefficient (ICC), and agreement between the two modalities for the previously listed findings as well as gallbladder wall thickness and common bile duct diameter was also assessed by ICC (17, 18) using PROC MIXED in SAS for Windows v. 9.4 (SAS Institute, Inc., Cary, NC). Models for each pairwise comparison were fitted with the outcome as the dependent variable and method of diagnosis (US vs. CT, US vs. final clinical diagnosis, or CT vs. final clinical diagnosis) as the fixed effect with dog identity included as a random effect (17, 18). The association between final clinical diagnosis and the diagnosis made by each imaging modality (US or CT) was assessed using separate logistic regression models in SAS for Windows v9.4 (PROC LOGISTIC), with final clinical diagnosis as the dependent variable. When data scarcity was an issue, penalized maximum likelihood estimation with the fifth option was used. An alpha level of 0.05 was selected *a priori*. Although no standard values for acceptable agreement using ICC have been established (19), Koo and Li suggested that ICC values less than 0.5, values between 0.5 and 0.75, values between 0.75 and 0.9, and values greater than 0.9 indicated poor, moderate, good, and excellent agreement, respectively. All statistical analyses were performed by board-certified veterinary epidemiologists (RW and KW).

## Results

Thirty-five patients were enrolled in the study. The age of the population ranged from 2 to 20 years old with a mean of 9.1 years old and a median of 10 years old. The weight of the population ranged from 2.6 to 50 kg with a mean of 12.4 kg and a median of 7.2 kg. The breeds included Yorkshire terriers (5), mixed breed dogs (5), Labrador retrievers (3), Chihuahuas (2), Miniature Poodles (2), Miniature Schnauzers (2), Standard Poodle (2), and one of each of the following: American Pit Bull, Beagle, Boxer, Cairn Terrier, Chinese Crested, Dachshund, English Bulldog, Jack Russell terrier, Maltese, Rat Terrier, Shetland Sheepdog, Shih Tzu, Welsh corgi, and West Highland White Terrier. This information is summarized in Table 1.

**Table 1:** Summary of patient signalment, biliary pathology (positive or negative), and final clinical diagnosis with cytology/histopathology (when available).

Age (years)	Sex	Breed	Weight (kg)	Biliary pathology	Described final clinical diagnosis and cytology/histopathology results
10	FS	Miniature Poodle	10.6	Positive	Gallbladder mucocele confirmed by surgery and histopathology. Liver histopathology—cholangiohepatitis
8	FS	Miniature Schnauzer	8.6	Positive	Copper storage disease on liver biopsy. Cholelith on imaging^*^
4	MN	American Pit Bull	33.5	Positive	Presumed anaphylaxis resulting in gallbladder wall edema
9	FS	Shetland Sheepdog	6.1	Positive	Gallbladder mucocele on surgery and histopathology. Liver histopathology—chronic active hepatitis
9	FS	Miniature Schnauzer	6.0	Positive	Pancreatitis. Presumptive cholangiohepatitis based on fluid analysis and cytology. Probable delayed plasma transfusion reaction.
20	MN	Mixed breed dog	6.3	Positive	Gallbladder mucocele confirmed by surgery and histopathology. Liver histopathology—cholestatic hepatic portal fibrosis and mild suppurative cholangiohepatitis
12	FS	Rat Terrier	8.8	Positive	Gallbladder mucocele confirmed by surgery and histopathology.
10	FS	English Bulldog	17.1	Positive	Gallbladder mass malignant carcinoid on histopathology
10	MN	Miniature Poodle	4.2	Positive	Pancreatitis causing extrahepatic biliary duct obstruction based on imaging and blood work findings including pancreatic lipase
12	FS	Chinese Crested	4	Positive	Histopathology—severe cholangiohepatitis (lymphoplasmacytic and suppurative) and severe acute hepatocellular necrosis. Concern for toxin then severe chronic pancreatitis^*^
6	FS	Maltese	5.7	Positive	Pancreatitis. Cholelithiasis. Liver cytology—cholestasis and suspect mild mixed inflammation with neutrophilic predominance
8	MN	Cairn Terrier	8.5	Positive	Liver histopathology—moderate-to-severe chronic cholangiohepatitis that is likely due to an ascending biliary tree infection with chronic cholestasis
10	MI	Chihuahua	3.4	Positive	Cholelith. Pancreatitis based on imaging and pancreatic lipase findings. Liver cytology—cholestasis
12	FS	Beagle	6.7	Positive	Ruptured gallbladder mucocele at surgery. Necropsy concluded systemic inflammatory response syndrome secondary to necrosuppurative cholecystitis
5	FI	Labrador Retriever	44.5	Positive	Cholelith. Chronic hepatitis. Liver cytology – significant cholestasis and mild to moderate hepatocellular vacuolization
9	FS	Mixed breed dog	2.6	Positive	Cholecystolithiasis, obstructive choledocholithiasis identified on CT, liver cytology—moderate hepatocellular vacuolization
9	FS	Mixed breed dog	18.2	Positive	Cholecystolith and gastritis based on CT and US. Hyperadrenocorticism. Liver cytology—mild lipid accumulation
10	MN	Standard Poodle	23.9	Positive	Bacterial cholangitis. Mineralized gallbladder sludge and pinpoint choleliths on CT. Bile cytology—significant bactibilia with *E. coli* growth. Liver cytology—no significant hepatocellular atypia
11	FS	Labrador Retriever	23.3	Positive	Severe pancreatitis with secondary extrahepatic biliary duct obstruction based on imaging and pancreatic lipase findings. Bile cytology—normal with no growth on culture. Liver cytology—cholestasis
10	FS	Yorkshire Terrier	7.2	Positive	Pancreatitis. Gastritis. Colitis. Mineralized gallbladder sludge/pinpoint cholecystolithiasis based on imaging, blood work, and pancreatic lipase analysis
9	MN	Yorkshire Terrier	4.9	Positive	Cholecystitis. Cholelithiasis. Pancreatitis. Normal bile cytology with no growth on culture. Liver cytology—compatible with lymphoma and evidence of cholestasis.
12	FS	Yorkshire Terrier	3.6	Positive	Extrahepatic biliary duct obstruction secondary to choledocholith and cholecystolithiasis. Surgery confirmed. Gallbladder culture—moderate growth of possible hemolytic *Escherichia coli*. Pancreatitis
11	FS	Maltipoo	4.7	Positive	Extrahepatic biliary duct obstruction secondary to choledocholith and cholecystolithiasis. Cholecystitis. Liver cytology—probable mild mixed inflammation and modest amounts of cholestasis. Bile culture—heavy growth of *Enterobacter cloacae*. Bile cytology—significant bactibilia
14	MN	Chihuahua	5.6	Positive	Cystic mucosal hyperplasia, mineralized gallbladder sludge/pinpoint cholecystoliths diagnosed on imaging. Right external iliac artery thrombus diagnosed on imaging. Liver cytology: Marked hepatocellular rarefaction. Bile cytology: normal. Bile culture: Growth of Bacillus sp.
2	FS	Mixed breed dog	15.2	Positive	Protein-losing enteropathy. Cholangitis vs. gallbladder wall edema. Pancreatic edema. Stomach histopathology—eosinophilic, lymphoplasmacytic gastritis with fibrosis, mild, chronic-active. Duodenum histopathology—eosinophilic, lymphoplasmacytic duodenitis with lacteal dilation, mild to moderate, chronic-active
12	FS	Dachshund	5.6	Positive	Mineralized gallbladder sludge or pinpoint choleliths. Concern for primary liver pathology. Liver cytology—minimal evidence of active chronic hemorrhage, mild hepatic lipidosis, probable mild mixed inflammation
5	MI	Labrador Retriever	50	Positive	Severe acute pancreatitis based on pancreatic lipase and fluid analysis. Focal choledochitis diagnosed on CT
12	MN	West Highland Terrier	10.3	Positive	Surgery—Gallbladder mucocele with possible rupture. Pancreatitis
4	FS	Yorkshire Terrier	3.4	Negative	Acute hemorrhagic gastroenteritis based on imaging, blood work, and clinical signs
4	FS	Yorkshire Terrier	7.2	Negative	Pancreatitis based on imaging and blood work including TLI, PLI, cobalamin, and folate^*^
14	MN	Standard Poodle	20	Negative	Cytology—Oral melanoma with liver metastasis
10	FS	Welsh Corgi	17.4	Negative	Histopathology—stage IV multicentric T-cell lymphoma of liver
3	FI	Jack Russell Terrier	5.6	Negative	Gastroenteritis, possible pancreatitis based on imaging, bloodwork, and pancreatic lipase analysis
4	FS	Shih Tzu	5.8	Negative	Presumptive Fanconi syndrome based on urinalysis and bloodwork including renal panel and leptospirosis testing
7	FI	Boxer	23.8	Negative	Liver histopathology—vacuolar hepatopathy (steroid hepatopathy), diffuse, chronic, severe with mild cholestasis. Small intestines histopathology—severe proliferative enteritis (causing clinical signs).

^*^Indicates CT and US were not performed on the same day due to dehydration concerns. CT, computed tomography; FI, female intact; FS, female spayed; MI, male intact; MN, male neutered; PLI, pancreatic lipase immunoreactivity; TLI, trypsin-like immunoreactivity; US, ultrasound.

No CT scans were excluded due to motion artifacts or other image quality issues. A total of 9 out of 35 (26%) ultrasound studies were incomplete in either missing images of the common bile duct or having images of insufficient quality to measure the duct size.

Six of 35 animals (17%) underwent sedation for ultrasound. Sedation protocols and doses were individualized but typically included a mix of dexmedetomidine (0.2–0.5 mcg/kg) and butorphanol (1–2 mg/kg) or methadone (0.5 mg/kg) administered intravenously or intramuscularly. A similar protocol, again at the discretion of the attending veterinarian, was used for all animals for CT.

Anorexia was the most common client-reported clinical sign (24/35 patients, 69%). Vomiting (21/35 patients, 60%) and lethargy (20/35 patients, 57%) were the next most common clinical signs. The least common clinical signs were jaundice (4/35 patients, 11%), owner-reported abdominal pain (2/35 patients, 5.7%), and fever (1/35 patients, 2.8%). Twenty-six of the 35 patients (74%) had at least two or more of the aforementioned clinical signs.

Of the 35 enrolled patients, 28 patients had confirmed biliary pathology and seven had no evidence of biliary pathology serving as controls. A summary of the patient signalment, presence of biliary pathology (positive or negative), and final clinical diagnosis with cytology/histopathology (when available) is provided in Table 1. The final clinical diagnoses of patients with biliary pathology included cholelithiasis (*n* = 13), gallbladder mucoceles (*n* = 6), cholecystitis (*n* = 21), gallbladder wall edema (*n* = 2), gallbladder wall mass (*n* = 1), CMH (*n* = 7), and extrahepatic biliary duct obstruction (EHBDO, *n* = 5). Of the cholecystitis patients, 9 of 21 were diagnosed on histopathology, 5 of 21 were diagnosed via cytology and culture, and 7 of 21 were presumptively diagnosed based on clinical and imaging findings. Fifteen patients had multiple diagnoses related to the gallbladder, including all the gallbladder mucocele patients that also had cholangiohepatitis and CMH. One of the gallbladder mucocele cases had confirmed gallbladder wall rupture at surgery with one other case having a possible rupture at surgery. Six other patients diagnosed with cholecystitis also concurrently had cholecystolithiasis as confirmed by twinkle artifact on US or mineral attenuation on CT. The five EHBDOs were due to pancreatitis (*n* = 2) and obstructive choledocholithiasis (n = 3). Mild gallbladder distention was commonly associated with gallbladder mucoceles and EHBDO. Three cases (two presumed cholecystitis and one CMH) also had cholecystolithiasis on the CT studies. The two gallbladder wall edema cases were a presumptive diagnosis based on one patient having hypoalbuminemia secondary to protein-losing enteropathy (with concurrent pancreatic edema) and one patient having presumed anaphylactic shock.

The final clinical diagnoses in the control patients included pancreatitis (*n* = 2), neoplasia involving the liver (*n* = 2, metastatic oral melanoma and lymphoma), hepatitis (*n* = 1), acute hemorrhagic diarrhea syndrome (*n* = 1), and presumed Fanconi syndrome (*n* = 1).

There was a significant association between the US imaging positive or negative diagnosis for biliary pathology and the final clinical diagnosis based on the full clinical workup, imaging, and cytology/histopathology if available (*p* = 0.0116) via logistic regression analysis with penalized maximum likelihood estimates. The odds of the final clinical diagnosis being positive when the US diagnosis was positive were 51.9 times greater than when the US diagnosis was negative. The overall calculated accuracy of the US imaging diagnoses compared to the final clinical diagnoses was 82.9% (29/35 of patients). The six false negative cases were all due to cholelithiasis identified on CT. When the six false negative diagnoses for cholelithiasis (defined as mineral attenuating material identified on CT and hyperechoic, distally shadowing structures on US) were removed, US agreed with the remaining 29 final clinical diagnoses. HU values on CT for cystolithiasis ranged from 218 to 412. No significant relationship was determined between the described variables and age, body size, or weight.

There was a significant association between the imaging CT positive and negative diagnosis and the final clinical diagnosis based on the full clinical workup, imaging, and cytology/histopathology if available for biliary pathology (*p* = 0.0022) via logistic regression analysis with penalized maximum likelihood estimates. The odds of the final clinical diagnosis being positive when CT imaging diagnosis was positive are 247.0 times greater than when CT imaging diagnosis was negative. The overall calculated accuracy of the CT imaging diagnosis compared to the final clinical diagnosis was 97.1% (34/35 of patients). One false positive case had identified a dilated common bile duct on the CT study (central portion of the common bile duct measured 5 mm in diameter), while the biliary tract was normal on US. This may have been due to transient dilation or secondary to sedative drugs used during CT as this patient was not sedated for the US examination.

**Figure 1:**
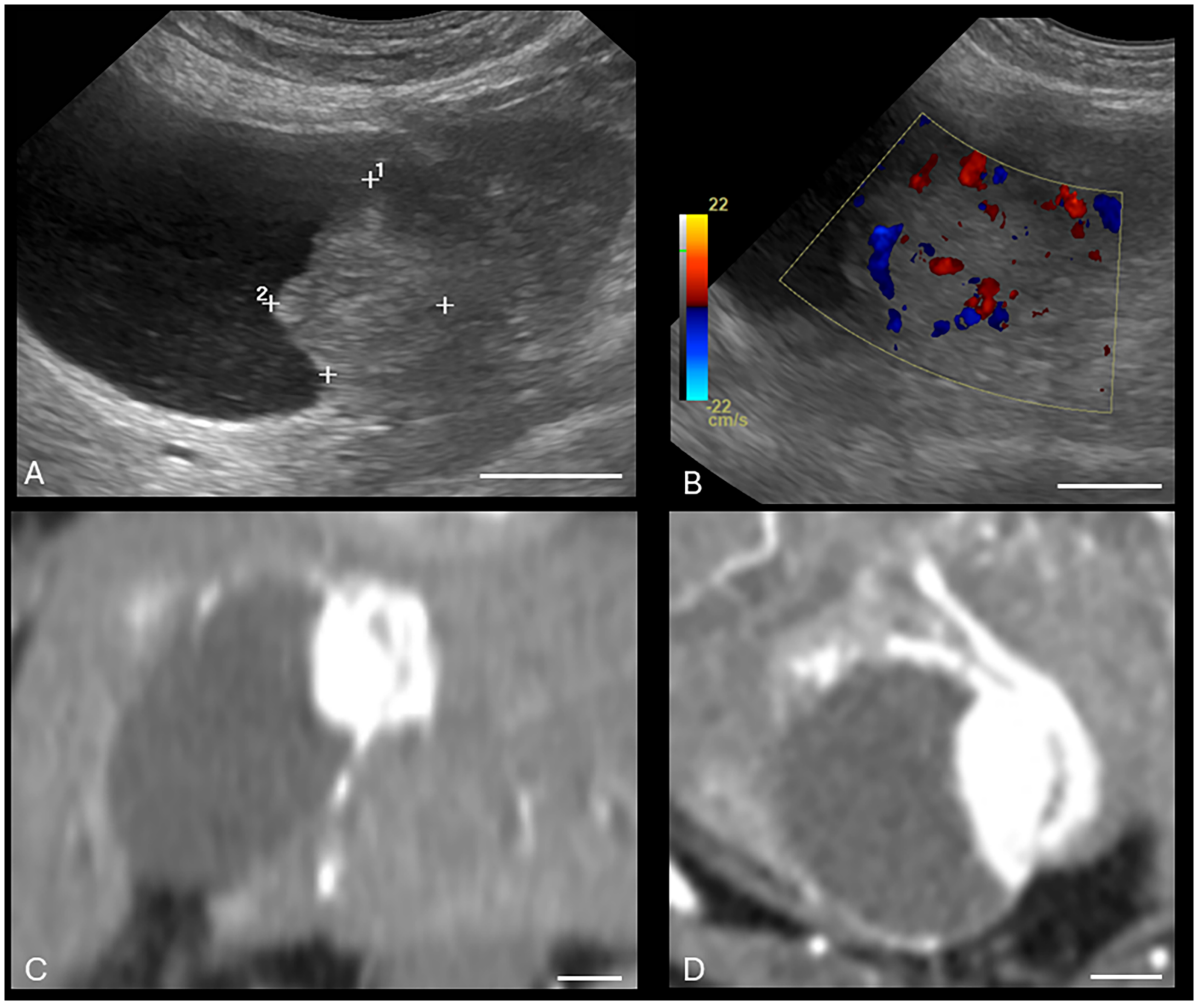
Canine malignant gallbladder carcinoid on CT and US. **(A)** Longitudinal B-mode ultrasound image of the gallbladder. There is an irregularly marginated, hyperechoic mass present within the gallbladder. **(B)** Same mass with color Doppler interrogation. The mass displayed a high vascularity on color Doppler interrogation. **(C)** Dorsal plane, soft tissue window, arterial phase post-contrast image (WW = 400, WL = 50) of the same patient. **(D)** Transverse plane, soft tissue window, post-contrast arterial phase (WW = 400, WL = 50) of the same gallbladder wall mass. The white calibration bar at the bottom right of each panel delineates 1.0 cm.

**Figure 2:**
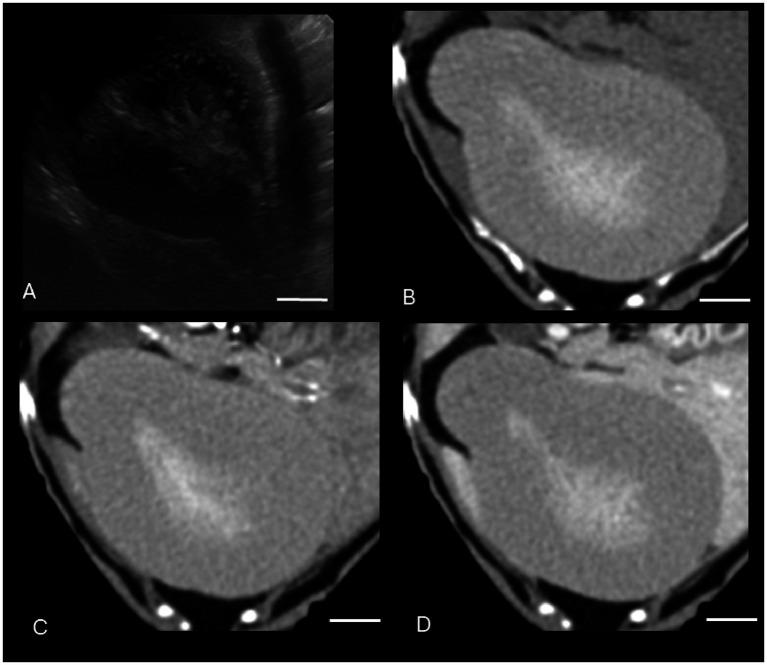
Canine gallbladder mucocele on CT and US. **(A)** Transverse B-mode ultrasound image of the gallbladder. There is central stellate hyperechoic intraluminal material, consistent with a gallbladder mucocele. There is additionally gallbladder dilation, a thickened and hypoechoic gallbladder wall, small volume surrounding peritoneal free fluid, and hyperechoic mesentery, consistent with peritonitis and concerning for gallbladder rupture. **(B–D)** Transverse soft tissue window (WW = 400, WL = 50) precontrast **(B)**, post-contrast arterial phase **(C)**, and venous post-contrast **(D)** CT images of the same patient. There is severe gallbladder dilation and central hyperattenuating material. The white calibration bar at the bottom right of each panel delineates 1.0 cm.

Both US and CT successfully identified the one gallbladder wall mass (malignant carcinoid) (Figure 1). Ultrasound and CT performed equally well in identifying all six gallbladder mucoceles, with no discordant pairs and an ICC of 1.0 (Figure 2). The imaging characteristics of all gallbladder mucoceles were consistent with those previously described (8–10).

**Figure 3:**
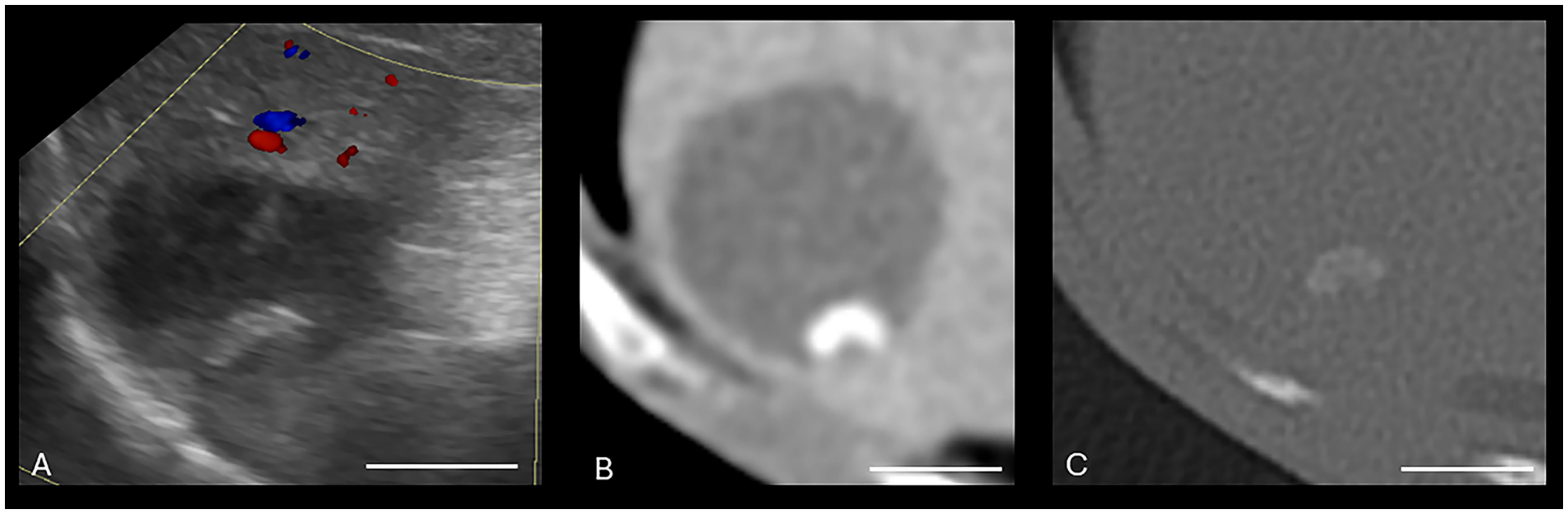
Canine cholelithiasis on CT and US. **(A)** Ultrasound image using compound imaging with color Doppler interrogation in the sagittal plane. This structure is hyperechoic and did not display a twinkle artifact during interrogation, so it was interpreted to be inspissated gallbladder sludge. The shadow along the cranial aspect of the structure was interpreted to be an edge shadow artifact. **(B–D)** Transverse bone window **(B)**, soft tissue window precontrast **(C)**, and soft tissue window post-contrast arterial phase **(D)** CT images of the same patient. There is a mineral attenuating structure in the gravity-dependent portion of the gallbladder with an HU of 287, consistent with a cholecystolith. The shape is similar between the two modalities. Non-contrast enhanced bone window (WW = 4,000, WL = 500). The white calibration bar at the bottom right of each panel delineates 1.0 cm.

There were various degrees of agreement between US and CT for the following conditions: gallbladder wall edema (moderate agreement), cholecystitis (good agreement), and CMH (poor agreement). Specific ICCs are provided in Table 2. Both US and CT successfully identified the two gallbladder wall edema cases. In addition, CT had three false positives for gallbladder wall edema. Both US and CT successfully identified the 21 cholecystitis cases based on symmetric or asymmetric gallbladder wall thickening, double-layered gallbladder wall, and hypoechoic gallbladder wall (14, 15). Many of these cases had concurrent pathologies such as a gallbladder mucocele and cholelithiasis. Ultrasound only successfully identified five of the 13 cases with cholelithiasis (Figure 3). Computed tomography identified all the cases of cholelithiasis with one additional false positive (a case with a histopathologically confirmed gallbladder mucocele).

**Table 2:** Final clinical diagnosis, number of cases, and interclass correlation coefficient (ICC) between ultrasound and CT diagnosis and between each modality and final clinical diagnosis for various biliary pathologies.

Final clinical diagnosis	Number of cases	ICC between US and CT	ICC between US and final clinical diagnosis	ICC between CT and final clinical diagnosis
Gallbladder wall mass	1	1.0	1.0	1.0
Gallbladder mucocele	6	1.0	1.0	1.0
Gallbladder wall edema	2	0.56	1.0	0.66
Cholecystitis	21	0.83	0.96	0.84
Cholelithiasis	13	0.47	0.70	0.96
Cystic mucosal hyperplasia	7	0.43	1.0	0.68

**Figure 4:**
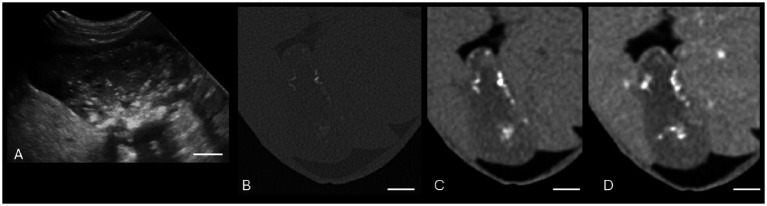
Canine cholelithiasis on CT and US. **(A)** Ultrasound image using compound imaging with color Doppler interrogation in the sagittal plane. This structure is hyperechoic and did not display a twinkle artifact during interrogation, so it was interpreted to be inspissated gallbladder sludge. The shadow along the cranial aspect of the structure was interpreted to be an edge shadow artifact. **(B–C)** Transverse bone window **(B)** and soft tissue window precontrast **(C)** CT images of the same patient. There is a mineral attenuating structure in the gravity-dependent portion of the gallbladder with an HU of 287, consistent with a cholecystolith. The shape is similar between the two modalities. The white calibration bar at the bottom right of each panel delineates 1.0 cm.

There was moderate agreement between US and CT in the measurement of gallbladder wall thickness (Table 3) when measured at the thickest point. Eighteen cases had a gallbladder wall that was thickened on at least one of the modalities. Eleven of these cases measured thickened on both CT and US, and seven cases measured thickened on only one of the modalities (Figure 4).

**Table 3:** Interclass correlation coefficient (ICC) and average difference between US and CT for measured values related to the biliary tract.

Measurement	ICC between US and CT	Average difference between US and CT (mm)
Gallbladder wall thickness	0.54	0.48
Common bile duct diameter	0.43	0.24
Hepatic duct diameter	0.67	0.61

There was poor agreement between US and CT in the measurement of common bile duct diameter. The common bile duct was identified in all CT studies. The common bile duct was identified in 18 of 35 (51.4%) patients on US; the remaining patients did not have any images of the common bile duct to review. Nine of the identified 18 common bile ducts on US were normal in size. Of the common bile ducts not identified on US that were identified on CT (17 patients), only four of these common bile ducts were enlarged on the CT studies, ranging from 3.5 to 9.9 mm in diameter. Sixteen cases had a common bile duct that was enlarged on at least one of the modalities. Nine of these cases measured enlarged on both CT and US, and seven cases measured enlarged on only CT. Of these 16 cases, the gallbladder was identified as enlarged in eight cases and normal in the other eight cases. Of the nine cases identified as enlarged on US, three were patients receiving opioid pain medication, and six were patients not currently receiving opioid medication.

The intrahepatic bile ducts were identified in four of the US studies (3 normal size and 1 dilated) and 20 CT studies (12 normal size and 9 dilated). There was moderate agreement between US and CT in the measurement of intrahepatic bile duct size. There was no statistical correlation relating the patient’s weight and age to the common bile duct and intrahepatic bile duct measurements when identified.

Hypoattenuating bile (as measured on precontrast CT) was identified in 11 cases with a range of 10–29 HU, as a previous study reported a median HU of 35.8 (range 11.2–48.6) in precontrast CT for dogs with no gallbladder sludge and a median HU of 39.7 (range 13.5–83) in dogs with sludge (9). The final clinical diagnoses of these cases included cholecystitis (*n* = 5), EHBDO (*n* = 4), gallbladder wall mass (*n* = 1), and a case with cholelithiasis, pancreatitis, and cholestasis. The only other instances of abnormal bile attenuation values were identified in the gallbladder mucocele cases with central hyperattenuating bile.

## Discussion

This study aimed to compare the performance of dual-phase abdominal CT to abdominal US when diagnosing biliary disease in dogs presenting with acute abdominal signs. The data supported the hypothesis that CT would allow for the detection of various canine biliary diseases with similar accuracy to US. Computed tomography identified common bile ducts in all patients, while abdominal US performed according to the Consensus Statement (7) only identified 51.4% of them. There also was no agreement on the identification of enlarged intrahepatic bile ducts between the two modalities. The results demonstrated a significant association between the imaging US and CT positive or negative diagnosis for biliary pathology and the final clinical diagnosis, indicating that both modalities performed well when comparing results to final clinical diagnoses.

There was perfect agreement between US and CT to identify gallbladder mucoceles and the single gallbladder wall mass (malignant carcinoid). This is not entirely unexpected, as CT features of gallbladder mucoceles have been previously reported (8–10), and the single gallbladder wall mass was strongly contrast enhancing on CT and displayed strong blood flow with color Doppler on US.

Surprisingly, there was also good agreement between the modalities to identify cholecystitis. Many of the features of cholecystitis, including symmetric or asymmetric gallbladder wall thickening and the presence of a double-layered gallbladder wall, were easily identified on CT (8, 11, 20). Similarly, wall thickening and a hypoechoic gallbladder wall were subjectively easily identifiable on US exam.

Cholelithiasis was the most common biliary pathology in this patient group which is similar to previous publications (8, 10). There was poor agreement between US and CT in the identification of intrahepatic and common bile duct cholelithiasis, with CT identifying cholelithiasis in 11 dogs and US identifying cholelithiasis in only five of these dogs. Cases not identified on US tended to be smaller choleliths (<2 mm). In 5 of 6 (83%) of these dogs, a small amount of gallbladder sludge was identified on US, but it was freely mobile and settled to the gravity-dependent portion of the gallbladder during the examination and so was considered incidental.

Both CT and US correctly identified the three cases of obstructive cholelithiasis. In people with biliary obstruction or cholecystitis, CT has been shown to be superior to US (21, 22). Of the six US cases that did not identify cholelithiasis, only one of the cases had a faintly mineralized structure within the region of the gallbladder on the corresponding abdominal radiographs. The decreased US identification of cholelithiasis was likely due to their small size, their decreased mineral composition causing a lack of US artifacts (distal acoustic shadowing and twinkle artifact), and the use of spatial compounding (23). In addition, there may have been movement of structures or superimposed gastrointestinal gas which limited visibility of the structures. A canine study comparing conventional US to spatial compound imaging evaluating canine nephrolithiasis found distal acoustic shadowing artifacts were present in 43% of spatial compound imaging mode and 86% of conventional imaging mode (23). It may therefore be beneficial to briefly interrogate the biliary system without spatial compounding to evaluate these small structures.

There was moderate agreement between US and CT for gallbladder wall thickness. Over half of the cases had a difference of >1 mm between US and CT measurements. It is plausible that this finding results from the difficulty on CT in differentiating true gallbladder wall from hyperattenuating bile. Most patients received both US and CT within 1 h of each other, but three (8.6%) patients were dehydrated upon presentation and had CT delayed by up to 24 h to allow for rehydration. It is possible that there was a change in pathology (progression or improvement) over this time period within these patients. If the underlying etiology was due to inflammation (as with pancreatitis or cholangiohepatitis), supportive care may have allowed the biliary pathology to improve due to decreased inflammation. In cases where EHBDO was present, pathology may have progressed as the obstruction persisted (11). In addition, intravenous fluid therapy may have led to a positive fluid balance leading to gallbladder wall edema from increased hydrostatic pressure or cholecystitis (8), especially in the three cases that had an overnight stay between US and CT exams. However, due to the small number of cases, this likely did not affect the overall average differences of the measurements. Volume averaging and magnification may also have affected the gallbladder wall thickness measurement on CT (24). Finally, different reference ranges may be needed when comparing gallbladder wall thickness on CT and US, as a recent study suggests that the upper limit in a dog without biliary disease on US is 1.30 mm (21), rather than 2.0–3.0 mm as reported with CT (12).

There was poor agreement between US and CT for common bile duct thickness. However, both US and CT correctly identified all three cases of EHBDO due to cholelithiasis and correctly identified all cases of dilated common bile ducts. Computed tomography has improved tomographic evaluation of the porta hepatis as compared to US which may allow for a more thorough evaluation of the common bile duct along its course (unfortunately, images of the porta hepatis were not available for all US exams). Distal acoustic shadowing from overlying gastrointestinal gas or mineral structures such as cholelithiasis and ribs, limited depth penetration, body confirmation, and limitations due to patient pain in the right cranial quadrant also likely limited the ability of US to evaluate these smaller structures in this region. In addition, the use of opioid pain medication in some of these patients may have resulted in dilation of the biliary system. Experimentally induced biliary obstruction may also behave differently to naturally occurring and more chronic obstruction identified in this population. Finally, different reference ranges may need to be applied for evaluating the common bile duct thickness on CT and US as prior studies have consisted primarily of smaller dogs (<15–20 kg), and the suspected normal range of <3 mm may be inaccurate for all patients (11, 25).

There was no agreement between US and CT in the measurement of intrahepatic bile duct size when identified. Intrahepatic bile ducts were the least identified biliary structure in both modalities, although more were identified on CT. Normally sized intrahepatic bile ducts may be too small to identify due to volume averaging (24). The ducts may not have been identified with saved images at the time of imaging due to operator error or inattention during the US examination as different individuals scanned different cases during this study. However, this likely mimics the clinical scenario as different clinicians will scan differently in emergency cases.

In this study, 11 patients exhibited hypoattenuating bile on CT. Hypoattenuating bile results from pathologies that impair the ability of the gallbladder to absorb fluid and concentrate bile, leading to increased fluid attenuation within the bile. The diagnoses of patients with hypoattenuating bile were consistent with previously reported conditions, including cholecystitis (bactibilia), EHBDO, and gallbladder wall masses (9).

The presence of gravity-dependent gallbladder sludge on US was not considered positive for biliary pathology, as while it has been linked to decreased gallbladder emptying, there has not been a strong correlation to clinical signs as an isolated finding in dogs (26).

The results of this study suggest that US and CT are both reasonable imaging choices when presented with a canine patient with acute abdominal signs suspected to be of biliary origin. Ultrasound is less expensive than CT and may be able to obtain more information in patients who are dehydrated and cannot receive intravenous contrast agents due to the risk of acute kidney failure. However, US is limited by the sonographer’s comfort level and skill and limitations of beam penetration to these deep structures. Numerous studies comparing CT to US for various other abdominal pathologies have shown improved CT identification or no significant difference between the modalities to identify intraabdominal pathologies including canine gastric neoplasia, canine surgical cases presenting for acute abdominal signs, and canine patients >25 kg for CT (1, 3, 27). In the current study, CT had the benefit of increased identification of mineral attenuating structures, along with the inherent benefits of increased tomographic evaluation of deeper anatomical structures and decreased study acquisition time. Computed tomography studies can be sent to a remote radiologist for interpretation which allows for a better global view of the pathology than US studies submitted for review by a sonographer, which tend to be more operator-dependent. This benefit of CT is often worth the tradeoff of potential adverse reactions of receiving intravenous contrast (although rare) and exposure to ionizing radiation as the images can be acquired more quickly, potentially hastening clinical decision-making. There is low risk for adverse events in the medical use of radiation for this type of CT study in dogs.

The main limitation of this study is the limited sample population and underpowering of this study, which may have resulted in a Type II error. A larger sample size would help to confirm these findings. The small sample size also made it challenging to evaluate the diagnostic performance between modalities of specific etiologies that were infrequently seen, such as gallbladder edema (*n* = 2) and neoplasia (*n* = 1). Another limitation is the lack of histopathology in all patients and within all regions of the biliary tract. Lack of histopathological diagnosis in all imaged regions may have revealed no true pathology in regions which appeared abnormal on the imaging modalities or confirmed pathology in anatomy appearing normal on imaging. This may be particularly relevant in the patient within this study where the common bile duct was dilated on the CT study but not on US. Finally, the bile was not sampled in all cases. Some additional biliary pathology may have been falsely classified as normal, resulting in false negatives, as it is possible for the gallbladder to look normal on both CT and US but show pathology on biliary cytology. However, it was not considered ethical to sample the bile in all cases given the small but real potential for serious side effects such as bile peritonitis.

Several technical factors may also have influenced the results of the study. For example, not all dogs were sedated for the US study acquisition, which may have limited the complete evaluation of the biliary tract given its subcostal location. The dogs in this study were under immobilizing intravenous sedation for the CT studies, as determined by the attending clinician, rather than general anesthesia or apneic conditions. Based on our experience with similar protocols in our hospital setting, we have found that these sedation methods generally do not result in significant motion artifacts affecting the biliary tract evaluation in CT; however, motion artifact could cause a limitation in the evaluation. In addition, the larger slice thickness in larger patients may have reduced the CT’s ability to detect smaller structures. However, this was considered necessary to balance the radiation dose and heat loading of the X-ray tube when scanning large patients. Specific to US, although the radiologists and residents in our hospital scan in a similar and complete manner following the abdominal US consensus statement (7), the savings of additional specific ultrasound images and cine loops were not standardized. The reviewed images were all considered adequate by the authors, but a further standardized protocol may have influenced the diagnostic performance of the US. Finally, the median weight of the population of dogs was 7.2 kg with the cohort consisting mainly of small- to medium-sized dogs. This may skew the agreement of accuracy as dogs > 25 kg may have pathology missed on US. However, the median body weight in this study represents what is faced in clinical practice and is similar to that of a recent study investigating cholangitis in dogs (28).

In conclusion, this study found variable, but generally good, agreement between CT and US in the diagnosis of biliary pathology in dogs presenting for acute abdominal signs. These findings suggest that CT may be able to be used in place of US if a sonographer is unavailable which may hasten decision-making (whether surgical or medical) in these patients, and CT may be superior in some diagnoses, particularly those involving cholelithiasis.

## Data Availability

The raw data supporting the conclusions of this article will be made available by the authors, without undue reservation.

## Author’s note

Abstract titled “Comparison of Abdominal CT to Ultrasound in the Diagnosis of Canine Biliary Disease Manifesting as Acute Abdominal Signs” presented at the 2021 ACVR Annual Conference with the preliminary data by SM.
